# Leptospirosis-associated pulmonary hemorrhagic syndrome: immune mechanisms, clinical manifestations, and experimental models

**DOI:** 10.1590/S1678-9946202668009

**Published:** 2026-01-30

**Authors:** Lara Rodrigues da Silva, Milena Carvalho Carneiro, Ana Carolina Mikejevs Lorga, Luana Barbosa Rodrigues dos Santos, Leonardo Moura Midon, Amaro Nunes Duarte, Thais Akemi Amamura, Lourdes Isaac

**Affiliations:** 1 Universidade de São Paulo, Instituto de Ciências Biomédicas, Departamento de Imunologia, São Paulo, São Paulo, Brazil; 2 University of Tennessee, Health Science Center, Department of Microbiology, Immunology and Biochemistry, Memphis, Tennessee, United States of America; 3 Universidade de São Paulo, Faculdade de Medicina, Departamento de Patologia, São Paulo, São Paulo, Brazil

**Keywords:** Leptospirosis, Complement system, Hemorrhagic pulmonary syndrome, Immunity, Leptospira.

## Abstract

Leptospirosis is a neglected zoonotic disease caused by bacteria of the genus *Leptospira*, mainly acquired via direct contact with water and soil contaminated by the urine of infected animals. This is most observed in tropical and subtropical regions, and it is strongly associated with urban population growth in areas lacking adequate sanitation conditions. *Leptospira* infection can lead to several clinical manifestations in humans, ranging from a nonspecific febrile illness to severe complications such as jaundice, renal failure, and life-threatening pulmonary disease. One of the most severe forms is leptospirosis-associated pulmonary hemorrhagic syndrome (LPHS), characterized by coughing, chest pain, dyspnea, and massive pulmonary hemorrhage. The mortality rate of LPHS is approximately 50%, with death generally occurring within 72 hours after symptom onset. The etiopathogenesis of LPHS remains poorly understood. Some studies suggest that *Leptospira* spp. may directly damage blood capillaries and alter vascular permeability. Additionally, the host immune response, via the cytokine release, high expression of adhesion molecules, and activation of the Complement System, may further disrupt endothelial integrity, promoting vascular leakage and the systemic dissemination of leptospires. Animal models are essential for a better understanding of *Leptospira* transmission, colonization, and pathogenesis. This review aims to consolidate current understanding of LPHS, with emphasis on its pathogenesis, immune mechanisms, clinical manifestations, virulence factors, and experimental models.

## INTRODUCTION

**Figure 1:**
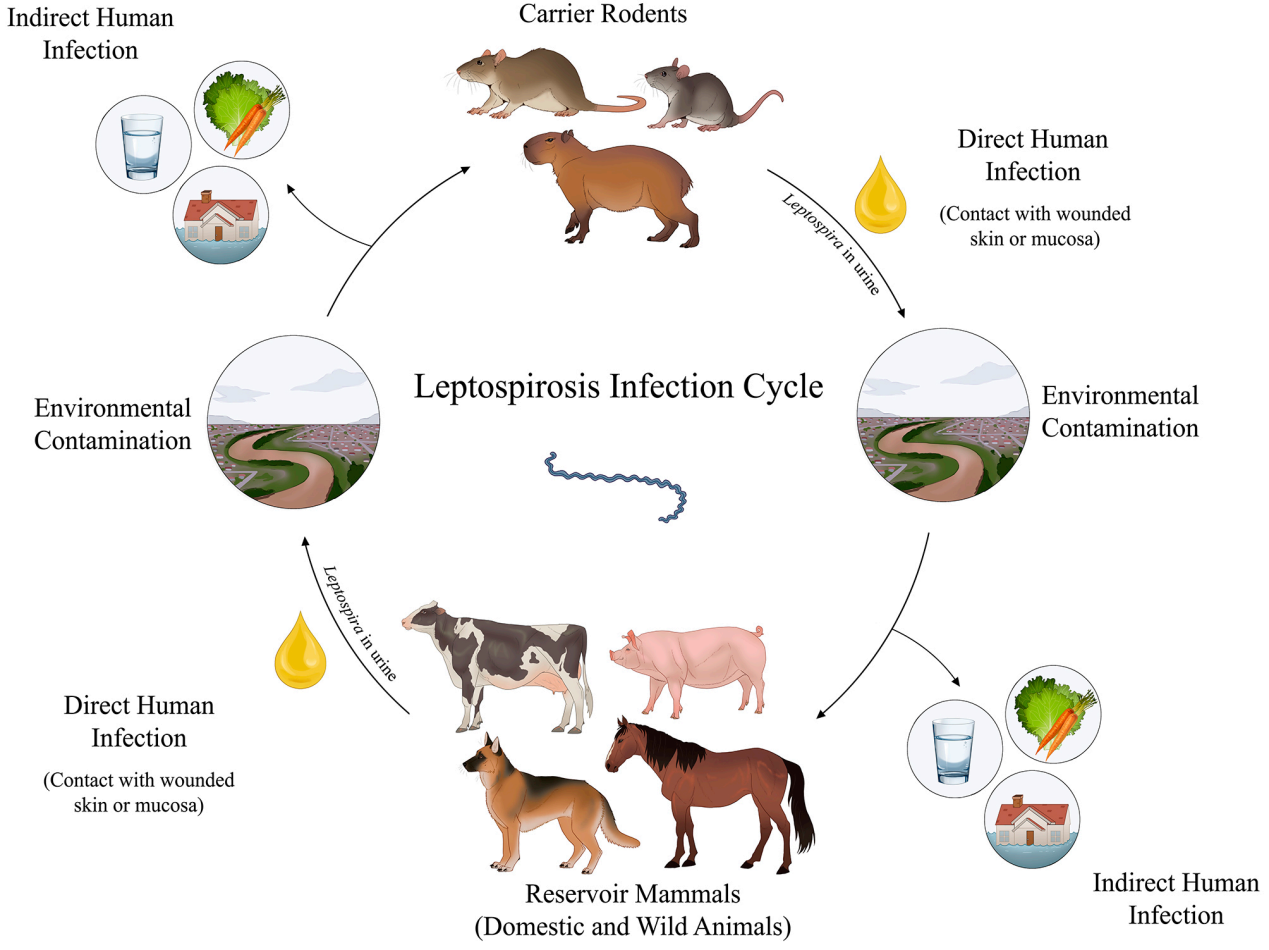
Infection cycle of leptospirosis. Dissemination by rodents: carrier animals—especially rodents—harbor *Leptospira* in their renal tubules and excrete them in urine for weeks or months. Direct transmission occurs via contact between infected rodents and humans or other animals, such as dogs, cattle, and horses. Indirect transmission happens via exposure to water and soil contaminated with the urine of carrier animals. Contamination of domestic animals: direct or indirect contact with contaminated sources. Once infected, these animals act as reservoirs and may further contribute to human infection and environmental contamination. Environmental contamination: flooded areas, agricultural fields, and natural water sources such as streams provide ideal environments for the spread of the disease. Human infection: occurs via the penetration of *Leptospira* via abraded skin or mucous membranes.

Leptospirosis is a re-emerging and neglected zoonosis caused by the pathogenic bacteria *Leptospira*, responsible for an estimated one million cases and 60,000 deaths annually worldwide^1^. Transmission is strongly associated with tropical and subtropical environments characterized by high humidity and inadequate sanitation, in which outbreaks often occur following natural disasters such as flooding^2^. The disease affects humans, wild, and domestic animals, in which urban rodents, particularly *Rattus norvegicus*, are the primary reservoirs due to their resistance to severe infection and their role in environmental contamination via urine^2^ (Figure 1).

**Figure 2:**
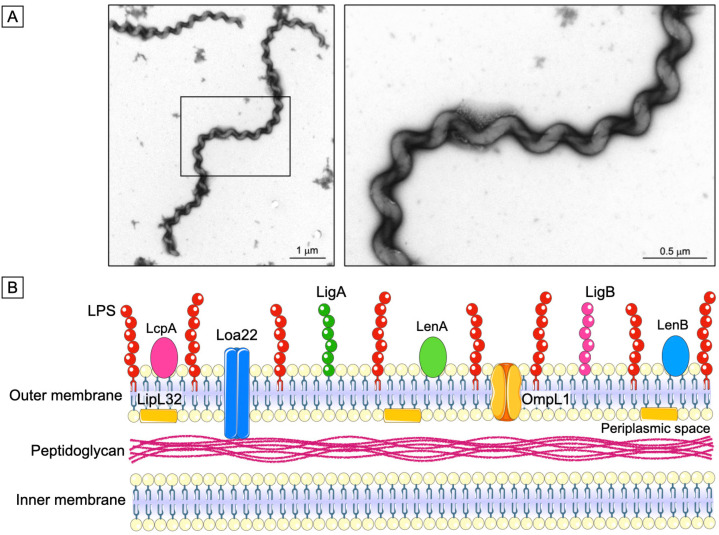
Structural representation of Leptospira: (A) Electron microscopy image of the spirochete *Leptospira*, illustrating its characteristic spiral shape; (B) *Leptospira* show a double-membrane architecture, comprising an inner membrane closely associated with the peptidoglycan layer and an outer membrane enriched with LPS and multiple surface-exposed proteins, including LipL32, LcpA, Loa22, LigA, LigB, OmpL1, LenA, and LenB.

Although leptospirosis is one of the most widespread zoonosis globally, it is still underreported due to nonspecific clinical symptoms and diagnostic challenges, particularly in resource-limited settings. *Leptospira* is a Gram-negative spirochete, with distinctive hooked ends and high motility conferred by periplasmic endoflagella (Figure 2). More than 69 *Leptospira* species have been identified and classified into saprophytic and pathogenic clades, with the latter being of particular medical importance^3^. Pathogenic species are further subdivided into serovars with distinct geographic distributions, which influence disease severity and immune response profiles.

These microorganisms have a double cell membrane, in which the inner is associated with peptidoglycan layers, and the outer contains lipopolysaccharides (LPS), phospholipids, and proteins. The components of the outer membrane are primarily responsible for the communication of these pathogens with the environment and the host. Moreover, the composition of the LPS in the outer membrane enables the classification of the bacteria into more than 300 different serovars^4^. Most infected individuals are asymptomatic or have only mild symptoms such as fever, headache, myalgia, nausea, and vomiting, which are often indistinguishable from other acute febrile illnesses^2^. However, 5% to 15% of these individuals may develop a severe form, called Weil's syndrome, which is characterized by multiorgan dysfunction involving the liver, kidneys, and lungs. Pulmonary involvement, including cough, chest pain, or mild hemoptysis, is observed in 20% to 70% of patients with severe disease^5^.

**Figure 3:**
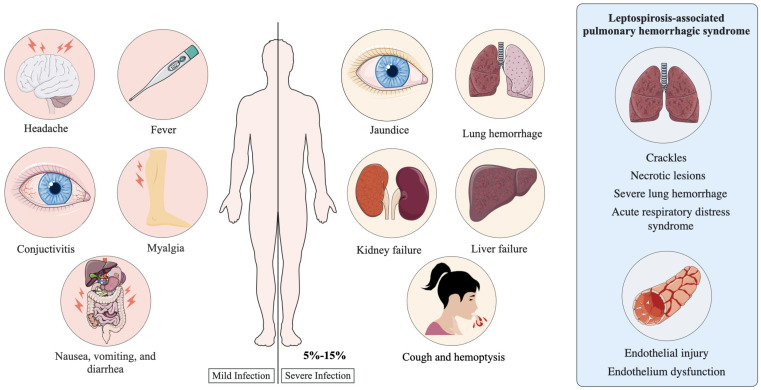
Symptoms associated with mild and severe leptospirosis. Mild cases are characterized by nonspecific symptoms, including fever, headache, conjunctival suffusion, myalgia, and gastrointestinal discomfort. Severe infections are marked by more serious clinical manifestations such as jaundice, acute kidney injury, hepatic failure, cough, and hemoptysis. Patients with leptospirosis-associated pulmonary syndrome (LPHS) may have pulmonary crackles, necrotic lesions, severe pulmonary hemorrhage, acute respiratory distress syndrome, and evidence of endothelial injury, and dysfunction.

The most critical pulmonary manifestation is Leptospirosis-Associated Pulmonary Hemorrhagic Syndrome (LPHS), defined by extensive hemorrhagic infiltrates within alveolar spaces, frequently leading to respiratory distress. LPHS has a mortality rate exceeding 50%, with death often occurring within 72 h of respiratory symptom onset^6-8^ (Figure 3). Although multiple factors — such as the bacterial burden and host condition — are known to influence disease progression, the immune mechanisms underlying LPHS pathogenesis remain poorly understood.

### Ethics

**Figure 4:**
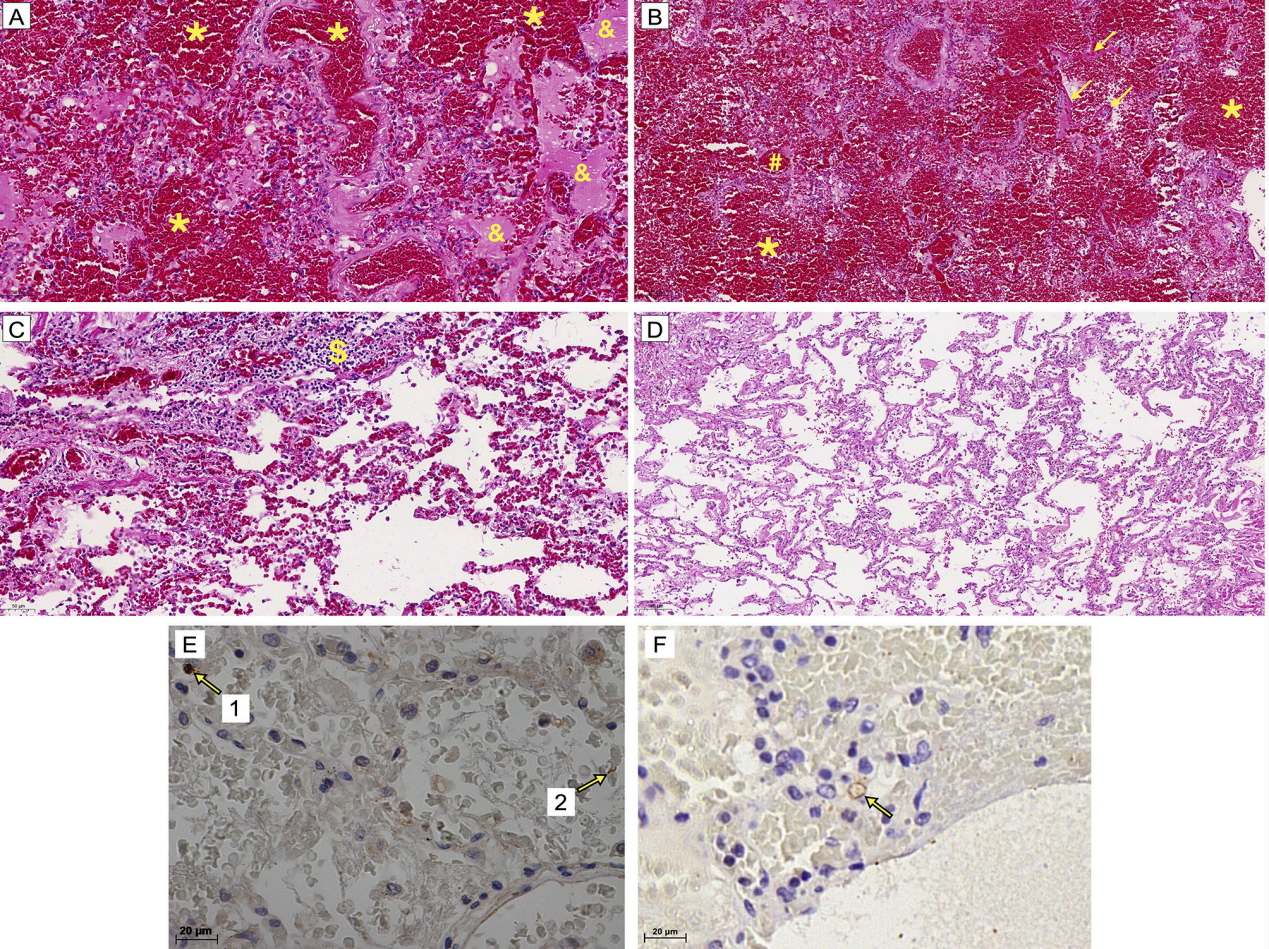
Photomicrographs of lung tissue stained with hematoxylin and eosin and *Leptospira* antigens detected by immunohistochemistry (IHC) in the lung tissue from a patient with LPHS. Samples from patients who succumbed to LPHS (A-C) were compared with those from a patient who died of myocardial infarction (D): (A, C) 200X magnification; (B, D) 100X magnification; *alveolar hemorrhage; ^&^edema; ^#^congestion; ^$^infiltrates in the alveolar space; arrows indicate hyaline membrane formation; (E) Arrow 1 highlights a positively stained immune cell, while arrow 2 indicates a preserved bacterial structure; (F) Arrow indicates the presence of *Leptospira* antigens. Magnification: 400X

Ethics approval of Figure 4: CAAE Nº 53237721.6.0000. 5467, classified as Exempt from CONEP Review.

### LPHS

The clinical signs of LPHS primarily involve alveolar hemorrhage and the development of Acute Respiratory Distress Syndrome^7^. Pulmonary symptoms typically appear between the fourth and sixth day of the illness^8^. Other severe manifestations may include renal failure, hepatic dysfunction with jaundice, systemic hemorrhage, thrombocytopenia, and hypotension—features often associated with poor prognosis^7^. However, these symptoms are not consistently present in all patients with pulmonary involvement, highlighting the clinical heterogeneity of leptospirosis^7,9^.

In 1998, one of the earliest studies on the topic described patients in Nicaragua who became infected after heavy rainfall and flooding^9^. These individuals developed cough, dyspnea, and hemoptysis, despite the absence of jaundice and renal manifestations^9^. However, pulmonary involvement in leptospirosis has been documented since the 1980s in diverse regions, including China^10^, Korea^11^, Brazil^12-14^, and other countries. These reports indicate that pulmonary manifestations of leptospirosis are neither geographically restricted nor serovar-specific.

Current evidence suggests that potential toxins from *Leptospira* spp. may contribute to vascular injury by compromising the integrity and permeability of capillary endothelium. Moreover, the immune response of the host, via the release of inflammatory cytokines and increased expression of adhesion molecules, may further exacerbate endothelial damage, promoting bacterial dissemination and vascular leakage^13^.

Spichler *et al*.^14^ investigated potential predictors of mortality in patients with severe leptospirosis in Sao Paulo, Brazil. The study identified several factors significantly associated with increased risk of death, including age over 40 years, thrombocytopenia (platelet count below 70,000/µL), oliguria (creatinine levels exceeding 3 mg/dL), and pulmonary involvement. Among these, pulmonary involvement emerged as the most critical predictor of poor prognosis.

Histological analyses of lung tissues from patients with LPHS revealed diffuse alveolar hemorrhage, pulmonary congestion, edema, fibrin deposition, and hyaline membrane formation^8,13^. Inflammatory cell infiltration within the alveolar spaces was also observed in some patients^13^ (Figure 4).

Interestingly, despite the extensive lung damage observed in patients with LPHS, the lung tissue contains a relatively low load of leptospires — mainly located within alveolar macrophages and septal capillaries — and fewer antigenic deposits compared to liver and kidney tissues^15^. This suggests that the histopathological changes observed in the lungs may be more closely linked to the action of circulating toxins produced by the pathogen rather than direct bacterial invasion (Figure 4).

### Immune response in LPHS

LPHS shows key similarities to other diseases characterized by dysregulated inflammation, particularly those involving elevated levels of pro-inflammatory cytokines^16^. Although the association between plasma TNF-α levels and disease severity has been known for some time, the precise role of this and other cytokines in the LPHS pathogenesis is unkown^17^.

Reis *et al.* ^18^ compared cytokine concentrations in patients with severe and mild forms of the disease and found that increased levels of TNF-α, IL-6, IL-8, and IL-10 were associated with fatal outcomes. Moreover, Lindow *et al.* ^19^ reported that patients with severe disease show heightened activation of neutrophils possibly caused by the increased levels of pro-inflammatory cytokines such as IL-1β, TNF-α, IL-8, and IL-6 at the infection site. Pathogenic *Leptospira* can trigger NETs formation^20^, which, while antimicrobial, may worsen inflammation and suggest a role in LPHS-related tissue damage^21,22^.

In addition to neutrophil involvement, an *in vitro* study by Gaudart *et al.* ^23^ showed that *L. interrogans* binds to the dendritic cell-specific intercellular adhesion molecule-3-grabbing non-integrin (DC-SIGN, CD209), stimulating the production of TNF-α and IL-12 (IL-12p70). Other *in vitro* studies have shown that the pathogenic *Leptospira* strains can adhere to and invade human macrophages, evading phagocytosis and inducing apoptosis and necrosis of these cells^24,25^. These findings suggest that the phagocytic response is impaired during infection and the systemic invasion of bacteria.

Bernardi *et al.* ^26^ observed increased expression of intercellular adhesion molecules (ICAM-1) and vascular cell adhesion molecules 1 (VCAM-1) in lung tissues from LPHS patients, compared to those without infectious or with non-leptospiral hemorrhagic conditions. These findings suggest that leptospiral infection promotes endothelial cell activation, enhancing neutrophil adhesion and contributing to local inflammation^27^. Supporting this, Sato and Coburn^28^ showed that pathogenic leptospiral strains upregulated ICAM-1 and ICAM-2 expression in human endothelial cell lines *in vitro*.

Since endothelial cells seem to be affected during pulmonary involvement in leptospirosis^26,29^. Bernardi *et al.* ^26^ investigated receptor expression in pulmonary vessels and alveolar septa from LPHS patient samples, which revealed that LPHS patients had higher TLR2 expression in pulmonary vessels compared to normal controls. Additionally, elevated expression of the Complement receptor C3aR was observed in the alveolar septa of LPHS lungs compared to non-hemorrhagic or non-infectious hemorrhagic lungs^26^. When C3aR binds to the C3a fragment, it can modulate VCAM-1 expression, promote the infiltration of immune cells into activated tissue, alter vascular morphology, increase vascular permeability, and contribute to tissue damage when excessively overexpressed^30^. Similar mechanisms probably occur in leptospirosis patients.

Moreover, studies in human patients have detected immunoglobulins (IgM, IgG, and IgA) and C3 along the alveolar septa of patients with LPHS^31^. A linear deposition pattern of these immune components was observed on the surface of type I and II pneumocytes in 18 out of 30 evaluated LPHS patients examined. This immune complex deposition is believed to compromise the integrity of the epithelial barrier, leading to necrosis and hemorrhagic manifestations. Similarly, a study involving 22 guinea pigs with LPHS also identified the deposition of immunoglobulins and C3 in the alveolar septa of 19 of these animals^32^.

Interestingly, the deposition of C3 in the alveolar septa of hemorrhagic lungs has been associated with the presence of immunoglobulins^31,32^, suggesting intense activation of the Classical Complement Pathway. This pathway is typically initiated by the binding of the C1 complex (C1q, C1r2, C1s2) to IgM and IgG, but can also be triggered by direct binding of C1q to pathogens, such as leptospires, or apoptotic cells^33,34^.

Additional studies have identified a correlation between the presence of anti-cardiolipin antibodies and the severity of leptospirosis^35^. Cross-reactivity between these antibodies and endothelial cells contributes to immune complex deposition and subsequent tissue injury^36^. The presence of these antibodies is associated with antiphospholipid antibody syndrome, which is associated with pulmonary capillary damage, intra-alveolar hemorrhage, and acute respiratory distress syndrome^37^. Furthermore, severe leptospirosis cases with pulmonary involvement have been associated with an exacerbated humoral immune response, with affected patients displaying higher antibody titers^38^.

The presence of C3, C3aR, and immunoglobulins, along with increased expression of adhesion molecules, supports the hypothesis that the immune system, particularly the Complement System activation, may significantly contribute to the exacerbation of hemorrhagic symptoms, influencing vascular permeability and promoting endothelial damage. Further investigation into bacterial virulence factors such as toxins and a comprehensive characterization of the immune response in LPHS are essential to improve our understanding of the disease.

### Potential virulence factors involved in LPHS

As previously mentioned, pulmonary analyses of patients with LPHS indicate a loss of endothelial integrity and disruption of intercellular junctions^7^. The ability of pathogenic leptospires to invade the host by adhering to epithelial and endothelial cells, as well to components of the extracellular matrix, is well-documented^39,40^. Martinez-Lopez *et al.* ^39^ demonstrated the *in vitro* ability of both *L. interrogans* Canicola and Copenhageni to compromise endothelial integrity. Evangelista *et al.* ^40^ provided evidence of *in vitro* interactions between leptospires and vascular endothelial cadherin (VE-cadherin) in human endothelial cells. Additionally, Eshghi *et al.* ^41^ demonstrated the capacity of *L. interrogans* to bind *in vitro* to human epithelial cadherin (E-cadherin) and VE-cadherin. Cadherins are responsible for maintaining cell integrity and adhesion^40,41^, and the interaction of leptospires with these molecules may contribute to the tissue damage observed in LPHS.

Sato and Coburn^28^ observed that, following *in vitro* infection of human endothelial cells with pathogenic and non-pathogenic strains of *Leptospira*, the pathogenic strain induced the disruption of adherens junction proteins. The bacteria affected the structures of VE-cadherin, p120-catenin, α-catenin, β-catenin, and actin filaments. Disruption of the tight junction protein Zonula Occludens-1 (ZO-1) was also reported. Tight junction proteins regulate paracellular traffic and plasma fluid movement into extravascular spaces, playing essential roles in various functions of epithelial and endothelial barriers under both physiological and pathological conditions. Dysfunction of these proteins in pulmonary tissue is often associated with acute lung injury and acute respiratory distress syndrome^42^.

Challa *et al*.^43^ investigated the role of *Leptospira interrogans* lipopolysaccharide (LPS) in the pulmonary hemorrhagic form of leptospirosis by testing the protection conferred by monoclonal antibodies (MAbs) in guinea pigs. The authors showed that LPS-specific Mabs that can agglutinate the bacteria (L8H4 and L9B11) provided complete protection against fatal pulmonary hemorrhage after challenge with the virulent Copenhageni strain, whereas a non-agglutinating MAb (L4C1) did not offer protection. These findings underscore the critical role of leptospiral LPS as a key antigen in pulmonary involvement, potentially mediating endothelial and epithelial activation and dysfunction that enhance alveolar-capillary permeability and contribute to intrapulmonary hemorrhage; nevertheless, further research is necessary to comprehensively elucidate these underlying mechanisms.

Campos *et al.* ^44^ demonstrated that infection of 3-D A549 spheroids with *Leptospira interrogans* triggers a distinct pattern of chemokine secretion, particularly inducing CXCL5 and CCL2. CXCL5 was consistently elevated, suggesting an important role in the early recruitment of neutrophils to the alveolar space. Moreover, CCL2 (MCP-1) was exclusively induced in the 3-D model, mirroring findings in leptospirosis-susceptible mice in which delayed CCL2 expression correlates with severe pulmonary involvement and tissue damage.

The detection of *Leptospira* antigens in the lungs of patients further suggests that pulmonary pathology in LPHS may be triggered by the presence of this pathogen and/or its toxins in the organ, which could initiate or amplify local chemokine-driven inflammation and immune cell recruitment. Taken together, these findings indicate that lung epithelial cells actively participate in the inflammatory response to *Leptospira*, producing chemokines that can orchestrate leukocyte infiltration and amplify endothelial–epithelial dysfunction.

Studies on human renal epithelial cells have reinforced the ability of pathogenic *Leptospira* in affecting the cellular junction complex^45^. *Leptospira* induces the degradation of p120-catenin and p0071, which are essential proteins for stabilizing E-cadherin at the cell membrane^46^. Furthermore, the bacterium disrupts tight junction proteins such as ZO-1, occludin, claudin-10, and cingulin^45^.

These studies suggest that the interactions of the bacterium and its toxins with endothelial and epithelial cells may be responsible for the dysfunction of intercellular junctions, as well as damage to septal capillaries, and increased tissue permeability. This process may enhance the release of pro-inflammatory mediators and lead to necrosis of pulmonary epithelial cells^7,13^.

### Experimental models of LPHS

As previously highlighted, leptospirosis in humans shows a wide clinical spectrum, with pulmonary hemorrhage representing a severe and often fatal complication. Developing appropriate animal models has been particularly challenging due to the inherent resistance of most rodent species to *Leptospira* infection. Overcoming this limitation is crucial to understand disease pathogenesis and develop effective therapeutic strategies. To address this, researchers have employed various wild-type and immunodeficient mouse strains—particularly those lacking Toll Like Receptor (TLR) 2, TLR4, MyD88, decay-accelerating factor (DAF)-1, or B lymphocytes^47,48^—as well as transgenic mice, to better understand host-pathogen interactions and disease mechanisms^49^.

Despite their usefulness, these alternative models pose several challenges, including higher costs and complexities in interpreting results. Studies in adult mice have demonstrated that disease progression factors depend significantly on the infecting *Leptospira* serovar, inoculum dose, and route of infection^50^. Golden Syrian hamsters are frequently preferred due to their acute clinical manifestations, which closely mimic those observed in human leptospirosis. This model is widely employed to assess strain virulence, determine infectivity, and evaluate infection routes^51^.

Guinea pigs have also been used as a LPHS model due to their susceptibility to pulmonary involvement. Nally *et al.* ^32^ reported extensive hemorrhagic lesions in the lungs and peritoneal surfaces following infection, identifying these as the primary causes of mortality. Rats and most mouse strains are generally unsuitable models for acute lethal leptospirosis, as they are resistant and show clinical manifestations only during a brief period after birth, specifically before three to four weeks of age. Beyond this period, these animals no longer succumb to infection. This observation suggests that the maturation of the immune system, which occurs by approximately five weeks of age in mice, is essential for controlling leptospirosis and ensuring survival^52^.

Notably, four-week-old C3H/HeJ mice, which are deficient in TLR4 signaling, show pulmonary hemorrhage and succumb to infection seven to eight days after exposure to serovars Icterohaemorrhagiae^52,53^, and Copenhageni^54^. However, they also develop resistance after this period, coinciding with immune system maturation^52^. This murine model has been used in identifying key factors involved in the host response to *Leptospira* spp.^45^. However, high doses of leptospires (>10^6^) must be administered to reproduce the clinical manifestations of the acute phase of the disease. In contrast, hamsters are susceptible to inoculations of only 10^2^–10^5^ leptospires^48^.

*In silico* studies based on the proteogenomics of *L. interrogans* aim to elucidate virulence factors, particularly virulence-modifying (VM) proteins, implicated in clinical manifestations. These studies have provided the basis for experimental designs that investigate the role of these proteins in leptospirosis pathogenesis^55^. The expression of genes encoding VM proteins is regulated both *in vitro*, under conditions that simulate the host's internal environment, and *in vivo*, using small animal models of acute infection^55,56^. These investigations have enhanced our understanding of leptospiral pathogenic mechanisms, including the potential link between hemolysis and pulmonary hemorrhage.

Despite the substantial progress achieved via *in vivo* and *in vitro* studies^54,55,57^, experimental models of leptospiral infection—particularly in mice—remain limited (Table 1). Further research is needed to develop and refine models that more accurately reflect the pathophysiology of LPHS.

**Table 1:** Pulmonary hemorrhage induced by *Leptospira* infection in various strains of mice

Article	Lineage	Sex	Age	Leptospira dose/Strain	Histopathological findings
Lehmann *et al*.^55^	C3H/HeJ	NI	3-6 weeks	5 × 10^7^ *L. interrogans* serovar Icterohaemorrhagiae Strain 11435	Lung and kidney lesions, arterial changes, inflammation, congestion, hemorrhage, and fibrin clots
Bandeira *et al*.^57^	C57BL/6 WT	NI	NI	10^3^-10^7^ *L. interrogans* serovar Copenhageni	Survived without clinical symptoms at all doses
	C57BL/6 Rag1 KO			10^3^- 10^7^ *L. interrogans* Copenhageni	Severe leptospirosis with jaundice, pulmonary hemorrhage in 10^6^ and 10^7^ doses
	C57BL/6 iNos KO			10^3^ - 10^7^ *L. interrogans* serovar Copenhageni	Survived without clinical symptoms at all doses; low susceptibility to interstitial nephritis
	CB17/SCID			10^6^, 10^7^ *L. interrogans* serovar Copenhageni	Severe leptospirosis with jaundice, pulmonary hemorrhage, and high leptospiral loads (inoculated with 10^7^)
	BALB/c				No clinical symptoms of leptospirosis
Silva *et al*.^54^	BALB/c	NI	NI	10^7^ *L. interrogans* serovar Copenhageni strain FIOCRUZ L1-130	Alveolar septum thickening, edema, and the presence of leukocytes and red blood cells in alveolar sacs
	C3H/Pas				Moderate thickening of the interalveolar septum
	C3H/HeJ				Severe alveolar hemorrhage; leukocyte infiltration and fragmentation of the interalveolar septum

NI = Not informed.

### Current challenges and knowledge gaps in LPHS

**Figure 5:**
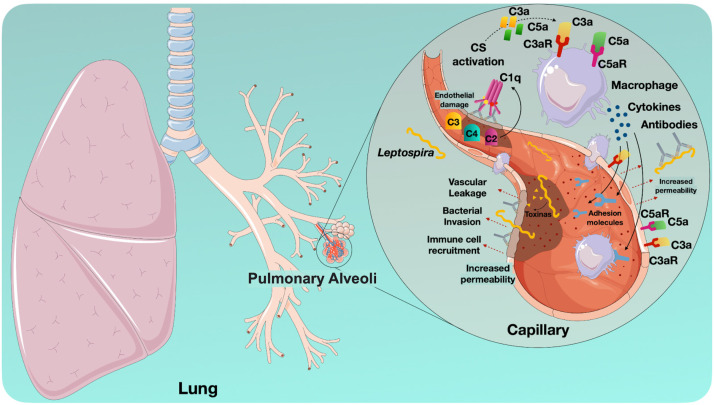
Schematic representation of the potential events leading to tissue damage in the lungs of patients with LPHS. *Leptospira* reaches the lungs through the bloodstream, where bacterial toxins potentially cause direct damage to endothelial and epithelial cells, disrupting tissue integrity and increasing vascular permeability. This disruption facilitates blood leakage and permits further entry of both bacteria and inflammatory mediators. Tissue damage can activate the Complement System via the Classical, Alternative, or Lectin Pathways. Anaphylatoxins, such as C3a and C5a, are released as a result of Complement protein activation. They interact with leukocytes and endothelial cells via anaphylatoxin receptors (C3aR or C5aR1/C5aR2). These interactions promote leukocyte activation and subsequent release of pro-inflammatory cytokines and mediators. These events amplify the inflammatory process, stimulate the expression of adhesion molecules, and increase vascular permeability. Excessive activation can exacerbate tissue.

Despite the advances in understanding leptospirosis, limited knowledge of the pathogenic mechanisms of *Leptospira* spp. hampers the identification of specific risk factors and virulence determinants that distinguish LPHS from other clinical manifestations of the disease. Furthermore, research on the disease is significantly hindered by its disproportionate impact on neglected populations, in which inadequate healthcare infrastructure and limited funding restrict both investigation and the advancement of new knowledge (Figure 5).

A critical challenge in leptospirosis management is the difficulty of early diagnosis, which contributes to poor outcomes^58^. The initial clinical presentation is nonspecific—characterized by fever, headache, and myalgia—and overlaps with other febrile illnesses^2^. This similarity frequently leads to misdiagnosis, delaying appropriate treatment and increasing the risk of progression to severe forms of the disease, such as LPHS. Additionally, traditional serological methods, such as the Microscopic Agglutination Test (MAT), may fail to detect low levels of specific antibodies during the acute phase. Although bacterial culture remains the definitive diagnostic method, it requires prolonged incubation periods, often spanning several weeks, making it impractical for timely clinical decision-making. Molecular methods, such as PCR, have the potential to significantly improve rapid detection^58^; however, their accessibility remains limited in resource-constrained endemic regions, which represents a major barrier to effective disease management.

The lack of reliable biomarkers for predicting leptospirosis severity limits the development of targeted therapeutic strategies. Currently, the treatment of severe leptospirosis primarily relies on respiratory support with mechanical ventilation with strategies for acute respiratory distress syndrome; early hemodialysis, to treat the acute kidney injury, adjusting the nitrogenous waste products (urea), fluid and electrolytes homeostasis; and antibiotics against *Leptospira* (i.e.: penicillin, ceftriaxone)^8,59^. However, the urgent need for adjunctive therapies remains unmet. Furthermore, identifying specific bacterial virulence factors associated with disease progression is crucial. Recent studies suggest that VM proteins may play a role in the pathogenesis and severity^56,60^.

Additionally, there is a lack of research on LPHS in both *in vivo* experimental animal models and human patients. No established animal model for investigating LPHS exists. This gap limits the investigation of disease mechanisms and the development of novel therapies.

Beyond clinical and experimental challenges, epidemiological surveillance of LPHS remains inadequate. The syndrome may be significantly underreported, particularly in non-endemic regions, leading to an incomplete understanding of its true prevalence and burden^1^. Addressing these challenges requires a multidisciplinary approach integrating improved diagnostic strategies, in-depth pathophysiological research, and enhanced epidemiological surveillance.

## CONCLUSION

The specific virulence factors and immune response mechanisms that drive LPHS remain inadequately characterized. A more comprehensive understanding of these elements could help overcome some of the major challenges associated with this condition. This knowledge may enable the identification of new therapeutic targets, such as leptospiral toxins, and facilitate the more effective prognostic and diagnostic tools based on immune response markers. Clarifying the mechanisms underlying LPHS could also aid in differentiating it from other leptospirosis manifestations, potentially contributing to earlier interventions and a reduction in case fatality rates.

## Data Availability

The complete anonymized dataset supporting the findings of this study is included within the article itself.
